# Using Mindfulness to Improve Mental Health Outcomes of Immigrant Women with Experiences of Intimate Partner Violence

**DOI:** 10.3390/ijerph191912714

**Published:** 2022-10-05

**Authors:** Ashley Vroegindewey, Bushra Sabri

**Affiliations:** Johns Hopkins University School of Nursing, Baltimore, MD 21205, USA

**Keywords:** intimate partner violence, immigrant women, mental health, PTSD, coping, mindfulness, mindfulness-based stress reduction

## Abstract

Immigrant women are disproportionately affected by intimate partner violence (IPV), which poses risk for mental health problems, such as PTSD and depression. Post-migration barriers limit immigrant women’s access to supportive services, which can further debilitate their mental health symptoms and their safety. The Being safe, Healthy, and Positively Empowered (BSHAPE) digital intervention was designed to address physical safety and healthcare needs of immigrant women through a multi-component approach that integrated mindfulness-based stress reduction (MBSR) practices. This paper reports qualitative feedback findings from eighteen Black immigrant women with recent IPV exposure and co-occurring mental health symptoms, who participated in the mindfulness sessions of BSHAPE. We identified elements of mindfulness that women perceived as beneficial in their healing. Women’s feedback indicated healing and empowerment through positive appraisals and coping strategies. The benefits were noted for mindfulness elements promoting self-compassion, self-actualization, intentionality of moving forward in life, and developing positivity or a sense of optimism. Other helpful elements were relaxation, self-care and reflection, self-awareness, self-control and focused thinking. Our findings show that incorporating mindfulness practices in interventions can be beneficial for promoting the healing and empowerment of immigrant women in abusive relationships.

## 1. Introduction

Immigrant women in the US, including immigrant women of African descent [1], are disproportionately affected by intimate partner violence (IPV). The friction between maintaining traditional cultural gender roles and new gender roles in Western society upon immigration contributes to greater vulnerability to IPV among immigrant women. For example, West African women face multiple instances of abuse post-migration and a sense of frustration with the existing options for assistance [2]. Other post-migration factors include partner or husband’s lack of employment and resulting feelings of disempowerment [3]. Stressors, such as language barriers, limited access to economic and community resources, limited or lack of knowledge of legal services and rights, adherence to traditional gender roles, social isolation and discrimination, and lack of cultural awareness by service providers, can place immigrant women at high risk for multiple health issues [1,2,3,4,5]. These barriers also inhibit immigrant women survivors from obtaining the appropriate trauma-informed help and attention that they need.

A traumatic experience, such as IPV, for marginalized women (e.g., immigrant) can greatly disrupt proper cognitive–emotion regulation and manifest as posttraumatic stress disorder (PTSD) or depressive symptoms [6,7]. In addition to mental health symptoms such as shock, confusion, guilt, self-blame, withdrawal, flashbacks, or insomnia, women manifest symptoms in the form of hypervigilance, avoidance, intense fear, or anxiety related to disclosure of IPV [8,9,10]. Traumatic life experiences, such as IPV also negatively impact the body’s stress regulation mechanism and immune response, which can develop into “toxic stress” and trigger a sequalae of various physical health problems, including an increased risk for morbidity [11,12]. The likelihood of stress becoming toxic and affecting women’s physical health is associated with immigrant women in abusive relationships facing continued and repeated IPV with a lack of options for breaking free from abuse. Immigrant women are at risk for continued and repeated exposures to IPV due to risk factors at multiple levels of the social ecology [13]. These include patriarchal cultural norms at the societal level, dependency on the abusive partner for immigration status at the relationship level, lack of community support or limited access to resources for support at the community level, and poverty, low education, poor mental health, social isolation, and lack of knowledge of resources at the individual level [13]. Factors such as social isolation and stigma related to IPV and mental health in immigrant communities can be barriers to seeking help for immigrant women [13,14]. The immigrant woman’s exposure to the first instances of abuse or violence offers no protective factors to another abusive incident if there are no supportive structures, culturally informed safety action plans, or strong coping mechanisms in place [13].

A chronic state of stress, fear, and poor mental health due to living in an abusive environment potentiate illness burden and the use of maladaptive coping strategies (e.g., substance abuse). The existence of comorbid health conditions inhibit women’s help seeking and leaving an abusive relationship [7,15]. Unaddressed needs for care may place immigrant women survivors of IPV at greater risk of harm and isolation rendered by IPV. Consequentially, effective mental health and stress management interventions are incredibly important to appropriately address the care needs of immigrant women with exposures to violence and trauma [6,7,15]. However, immigrant women with mental health problems face numerous barriers to accessing mental health care. These include language barriers or stigma associated with mental health or mental health counseling. Other barriers include dependence on an abusive partner, a controlling partner who restricts access to services, or lack of available culturally sensitive practices for mental health care [14]. Abusive relationships exacerbate these barriers for immigrant women. Mindfulness practices can be alternative approaches to support immigrant women with mental health problems who are unable to access stress management and mental health care services due to immigration, stigma, religious, cultural, socio-economic, or partner-related barriers.

Evidence shows that mindfulness approaches can lead to significant improvement in mental health [16,17,18]. Mindfulness is defined as “awareness of one’s moment-to-moment experience nonjudgmentally and with acceptance” [16] (p. 2). The practice of non-passive acceptance reinforces attention on the present moment experience whenever the mind wanders by shifting from trauma-related stimuli to the present experience [9,16]. Mindfulness practices encourage the individual to focus on and accept the consciousness or emotional experience rather than revert to negative coping mechanisms often associated with PTSD symptoms, such as suppression, over-engagement, numbness, or avoidance [9,16,19,20]. Attention control exercises, such as alternative breathing or open monitoring, train women survivors to be present in the moment with non-judgement and acceptance while simultaneously targeting positive cognitive reappraisal [16,19,21]. Possessing a nonjudgmental stance toward traumatic experiences encourages positive cognitive changes, such as desensitization, as well as decreased rumination and perceived stress [20,21]. Thus, mindfulness practices can lead to decreased symptoms of PTSD through improved emotion and stress regulation, increased attention, and non-judgmental acceptance [9,16,19].

Decentering, another component of mindfulness, helps in navigating trauma-associated negative emotions and alterations in arousal by increasing awareness of one’s sensory, cognitive, and affective responses that do not necessarily require exposure to one’s trauma [18,19,21]. Decentering one’s thoughts and emotions and perceiving them as passing sensations or mental events rather than identifying them to be accurate representations of reality increases metacognitive awareness and attention [19]. Decentering may predict better clinical outcomes, leading to lower rates of depressive relapses [16]. Thus, various components of mindfulness components can lead to improvement in mental health symptoms among immigrant women with experiences of IPV.

The impact of mindfulness in promoting the healing of IPV survivors can be explained by the Taylor and Aspinwall’s Psychosocial model (1996). According to this model, life stressors can indirectly affect psychosocial outcomes based on how the individual perceives or accepts the stressor and how an individual copes [22]. Appraisal is defined as cognitive evaluation and interpretation of an experience that influences the perception of the stressful experience [22]. Coping is the conscious and voluntary management of stress in response to the negative experience with the expectation to reduce stress and build resiliency [23]. Mindfulness-based stress reduction activities can enhance women’s internal strengths as resources and promote adaptive appraisals (e.g., positive self-actualization) and coping (e.g., relaxation), which can lead to positive outcomes (e.g., empowerment) and promote the path to healing. Such approaches can especially be beneficial for marginalized populations, such as immigrant women, who are unable to access mental health care due to stigma, shame, transportation, and economic issues [13,14]. Mindfulness-based approaches can be utilized in women’s homes daily without the need for an in-person visit to a provider. Thus, additional research is needed to build evidence for the beneficial effects of mindfulness approaches for marginalized women with mental health issues related to IPV and other stressors in their lives.

### Rationale and Significance

Although studies examined the impact of in-person mindfulness-based stress reduction (MBSR) approaches for improving mental health symptoms of diverse populations [9,18,20], including that of US-born survivors of interpersonal violence or IPV [17,19,21], studies are yet to explore the impact of remote phone-delivered mindfulness approaches on the mental health of immigrant women with experiences of IPV. Further, studies are yet to explore how elements of mindfulness delivered over the phone could promote healing among immigrant women with co-occurring IPV and mental health problems. Remotely delivered trauma-informed mindfulness can be a useful approach for addressing stress reduction and the mental health care needs of immigrant women in a stigma-free environment. The approach can be especially beneficial for women who voluntarily or involuntarily are unable to access mental health care services due to poverty, lack of insurance for mental health care, stigma, socio-cultural, religious, or other reasons. The format of remote delivery can be useful for women who face transportation barriers, or stigma and shame in sharing sensitive experiences face-to-face, as well as for those who are impacted by challenges related to situations such as the COVID-19 pandemic. Digital interventions, such as phone applications, coupled with safety strategies, have the potential to reach women who are unable to attend traditional in-person interventions due to fear or control of the abuser by providing privacy per the woman’s convenience and safety [24]. Using a sample of Black immigrant women, this study explored the impact of mindfulness in improving women’s mental health, as well as the components or elements of mindfulness that women perceived as beneficial in their healing. For this study, “Black immigrant” refers to foreign-born immigrant women coming from Africa or immigrant women of African descent. The findings can be informative for practitioners as to which elements to emphasize in mindfulness interventions for women with IPV exposures and those with comorbid PTSD symptoms or depression.

## 2. Materials and Methods

### 2.1. BSHAPE Design and Development

The Being Safe, Healthy, and Positively Empowered (BSHAPE) intervention was designed to address the physical safety and mental health needs of immigrant women through a multi-component approach that integrated mindfulness, or mindfulness-based stress reduction (MBSR), in its intervention. While BSHAPE addressed other needs through components such as strengths-based feedback, psychoeducation on HIV risk reduction, or education on immigration-related resources, the mindfulness combined with weekly behavioral activation homework assignments focused on stress reduction, mental health, and safety. The BSHAPE pilot trial focused on Black immigrant women because many Black women in the US migrate from conflict-affected regions, with some forced to flee their home countries as refugees [25]. Thus, they are more likely to have lifetime cumulative exposures to violence, such as war or the imminent threat of harm. However, the intervention was designed to be adaptable for immigrant women from other racial/ethnic backgrounds.

Components of the BSHAPE intervention included an assessment and participation in a phone-delivered strengths-based feedback session, followed by online psychoeducational modules and MBSR phone sessions with a facilitator. The details of the intervention are provided elsewhere [25]. The phone-based MBSR component of BSHAPE consisted of weekly half-hour sessions between the woman and a trained masters-level graduate student facilitator for four weeks. Facilitators of the mindfulness sessions were trained masters-level graduate students who received training in mindfulness using a MBSR curriculum. Facilitators completed two days of in-person or virtual training on MBSR and were given a standardized mindfulness curriculum manual with audio recordings of the meditation’s practices. The MBSR curriculum was designed by Tawanna Kane, a certified and experienced mindfulness instructor with experience working with diverse and underserved populations [26], and was an adaptation of Jon Kabat Zinn’s MBSR program [25,27].

The MBSR sessions were designed to be individualized, self-guided, and exploratory, prompting one to think about daily life stresses and triggers. The one-on-one sessions between mindfulness facilitators and women allowed open dialogue and expression of feelings, thoughts, and perceptions of safety and security in their own words, especially on sensitive topics, without them feeling like their privacy was invaded. The facilitators introduced different stress reduction techniques to the woman, which included alternative breathing methods, promoting weekly pleasurable activities, body scans, or “lovingkindness” meditations. The weekly sessions also allowed for the woman to express any concerns that she had, including weekly safety check-ins with the woman and discussing a safety plan in any event that the woman felt unsafe and provided information about supportive services. Upon completion of each session, facilitators documented the woman’s fidelity to the intervention’s activities and her response [25]. All study procedures were approved by the home institution of the study’s principal investigator (blinded for review).

### 2.2. Recruitment

BSHAPE research assistants recruited women via emails, listservs (subscribers on an electronic mailing list), flyers posted in community locations, study information posted periodically in the organization’s newsletters, social media platforms through a social media ad campaign, snowball sampling, and word-of-mouth throughout the United States through assistance from universities and colleges, student associations, immigrant/refugee agencies, or churches. Women who were interested answered the screening questions to determine eligibility on the study website. The research team reviewed eligible women’s registration information and contacted them by phone to obtain informed consent, to enroll them into the study, and to set up an appointment for their remote baseline survey and assessment. To be eligible, women had to be (a) 18 to 55 years of age, (b) a foreign-born immigrant woman born in Africa or a Black immigrant born in any other region outside the U.S., (c) in a current or past intimate partner relationship, (d) report lifetime exposures to violence, with clinically significant symptoms of PTSD (scores higher than 2.5) [25,28]; and/or depression (score of 10 or higher) [25,29] and (e) report at least one sexual HIV risk behavior (e.g., multiple sexual partners).

### 2.3. Participants

In the BSHAPE study, 42 out of 144 women reported recent (within past 12 months) experiences of IPV. This paper reports findings from 18 women randomized to the intervention arm of BSHAPE, who were recent IPV survivors and participated in the remote BSHAPE mindfulness sessions. IPV in this study included physical, sexual, and/or psychological abuse. The average age of women with recent IPV was 35.39 (SD = 8.96). A total of 47% (*n* = 8) were married, 17.6% (*n* = 3) were separated, and others were either in non-marital relationship or were partnered or divorced. Seventy-five percent (*n* = 12) were in a heterosexual relationship with two identifying as asexual and others in the bisexual or queer category. Approximately 44% (*n* = 8) were recent immigrants were in the US for four years or less than four years. Education was high, with most women having a bachelors or master’s degree (71%, *n* = 12). Most women (89%, *n* = 16) were born in Africa, followed by those born in other regions outside the U.S. Fifty percent (*n* = 9) indicated moderate or moderately severe depressive symptoms, with fifty percent of women reporting severe depressive symptoms *(n* = 9) and/or co-occurring clinically significant PTSD symptoms (sixty-seven percent, *n* = 12). Among lifetime trauma exposures, besides family violence or IPV, a large percentage of women reported experiences of collective identity trauma (e.g., racism and war experience) (89%, *n* = 16), personal identity trauma (i.e., events challenging the self, such as bullying and sexual abuse) (94.4%, *n* = 17), attachment trauma (e.g., abandonment by parent) (83.3%, *n* = 15) and survival trauma (83.3%, *n* = 15) (e.g., exposures to torture, accidents, or disasters) [30].

### 2.4. Measures and Data Collection

Qualitative data were collected and tracked from women weekly through fidelity forms. The fidelity forms included documentation of how the trained mindfulness facilitator rated the fidelity of each session to the script and core components of the MBSR intervention. Every week, the facilitator documented the women’s adherence to home practices, their perceptions of safety during safety check-ins, mindfulness components covered during the session, facilitator’s reflection, and the women’s evaluation. The forms documented the women’s evaluation of the overall session verbatim and their ratings of the overall quality of the facilitator’s delivery of mindfulness. Fidelity was also ensured through weekly supervision with the expert investigator on the team.

Adherence to the completion of the phone-based sessions and its weekly interventions were also tracked and documented as measurable outcomes. Upon completion of the four-week phone sessions, a feedback survey was given to the women, asking them to share their overall feedback (what was helpful, what they liked most, and if there was anything that could be improved or added), any barriers they faced in active, consistent participation, and whether they would continue the home practices beyond the four weeks. Additionally, women were asked about the needs for additional resources that would support them in continuing their practice.

### 2.5. Data Analysis

For this study, a qualitative descriptive approach [31] was used for comprehensively capturing immigrant women survivors’ experiences and stories in their own words. Data were analyzed using the qualitative content analysis procedure [25,32]. The process involved coding and categorizing the feedback into themes using the questions in the feedback guide, followed by additional coding of content not covered by the feedback guide. The analysis was conducted by two members of the research team (the principal investigator and the graduate student), with members agreeing to the content and meaning of each theme, summarizing based upon the frequency of each theme and refining them further into sub-themes. Pseudonyms were used to present participant’s quotes.

## 3. Results

Women shared how mindfulness practices were beneficial for their health and well-being. The key benefits were illustrated as healing and empowerment through positive appraisals and coping. The beneficial effects were noted for elements promoting self-love, self-actualization, intentionality of moving forward, and possessing a positive attitude towards life. Women also reported utilizing coping strategies, such as relaxation, self-care and reflection, self-awareness, self-control, and focus. In our analysis of quantitative data, women in the intervention arm also reported improvement in PTSD and depressive symptoms. The findings of the quantitative data are reported elsewhere [25].

### 3.1. Healing and Empowerment

The BSHAPE sessions were designed to enhance coping skills (e.g., nonjudgmental acceptance) to better counteract negative perceptions of stress, and to promote healing and positive mental health outcomes. Self-empowerment was the overarching theme found and can be defined as the state when the individual learns to “observe a feeling rather than be the feeling… With this comes a new internal freedom. The individual becomes less reactionary and can make a conscious choice in how to respond to internal states” [33] (p. 74). One woman described feeling “empowered” perfectly in her own way, while simultaneously representing how survivors of IPV can attain a sense of achievement, self-worth, and peace in life:

*[I feel] at peace, exhilarated…felt like I achieved a lot for quite some time, and felt like I deserved this. I feel at peace doing this as a reward*

(Tina, 32 years old).

### 3.2. Positive Appraisal

Positive self-appraisals were highlighted in survivors’ feedback on their engagement in mindfulness practices. Mindfulness practices promoted self-actualization, lovingkindness, intentions of moving forward in life, and developed positive attitudes.

#### 3.2.1. Self-Compassion or Lovingkindness

The “loving-kindness” meditative component of mindfulness promotes compassion or love towards oneself and others in addition to adapting a non-judgmental mindful existence [9,18]. Some survivors (*n* = 3) shared that acknowledging and accepting their past and present, without avoiding or self-condemning, helped them to develop a stronger sense of self-compassion and lovingkindness towards themselves and others. This is fundamental for women survivors of IPV, as many women struggle to show self-compassion without judgement, which could propitiate severe PTSD symptoms, such as avoidance [19,21]. One woman who was married reported finding peace in everyday moments, such as washing the dishes, because she realized that she could replace self-inflicted suffering with lovingkindness towards herself and others:

*Lovingkindness…is our way to do good, in world, first have peace within me, then show love to people; and everything follows. Love comes at end of everything. To reduce stress, love self. Don’t need to suffer any more*

(Sara, 38 years old).

#### 3.2.2. Self-Actualization

Some women survivors (*n* = 4) reported making significant self-realizations. They felt more affirmed in their self-worth and in their strengths and potentiality to do things beyond what they thought they were capable of before the mindfulness session. One woman who liked listening to music and prayer, said:

*I think it is a good thing to do lovingkindness meditation because it … allowed me to go back far away and see some good things from the past, it was a good session, and I liked to do it again; Closing my eyes and going somewhere, imagining to be somewhere and when I come back, the bell ringing; Feeling like I’m far away, made me think of a lot of things…that was helpful*

(Shahana, 36 years old).

Another woman survivor, who was a busy mother and aspiring nurse, expressed her potential in becoming a nurse:

*I don’t like seeing blood and people in pain; but it is a good job. Nurses are working. I can be good at it*

(Linda, 37 years old).

#### 3.2.3. Intentionality of Moving Forward

Some survivors (*n* = 4) decided to take a step further in moving beyond the suffering of pain and being under the control of their abuser. This re-appraisal of control in one’s own life proved to be a pivotal moment for many of the IPV survivors, as it empowered them to make choices or take opportunities that are better for themselves [8] and for others.

For example, one woman, who was married with two teenage children, recently immigrated to the US and was trying to obtain a green card and learn a new language. She was striving to make life easier for her children despite her own pain and loss:

*I am trying to work on my English and my marriage. I don’t want my kids to suffer or to have the pain*

(Cindy, 40 years old).

Another woman, who was married to an American citizen, expressed her intention in taking more control of her life by being true to herself through the practice of journaling:

*My intention is to not let other people control my life… I aspire to be truthful to myself…*

(Libby, 31 years old).

#### 3.2.4. Positive Attitude towards Life

Six women (*n* = 6) expressed developing a positive attitude, or a sense of optimism, from the mindfulness sessions. They viewed the sessions as beneficial and with an overall sense of feeling “positive” at the end of the sessions. This is contrary to what most survivors of IPV feel about themselves and their outlook on life, as most feel the despondency related to depression or PTSD [21]. From this change in perspective, some women were able to see some good things from the past, which propelled them towards a more optimistic approach to the present moment and future. One survivor who was a student and enjoyed the positive affirmations of the sessions, shared:

*It [mindfulness session] was just a positive experience; All those things that I was feeling… I was able to conquer with a more positive attitude and outlook*

(Karla, 22 years old).

### 3.3. Positive Coping

Women reported benefits of mindfulness sessions in terms of promoting engagement in coping strategies, such as relaxation, self-care and self-reflection, faith-based practices and prayer, self-awareness, and practicing self-control or focused thinking.

#### 3.3.1. Relaxation

Many women (*n* = 9) felt relaxed either mentally and/or physically after engaging in mindfulness practices. For most women, relaxation was a sense of letting go or emptying oneself from daily stress or worries that may be especially triggering for those with experiences of IPV. For some women, relaxation provided a sense of “calmness” or “peace”, especially with the body scans or alternative breathing exercises. One woman, who enjoyed listening to music and taking naps, demonstrated how relaxation lifted a mental load from her, freeing herself from worries. It also manifested itself physically:

*Mind is even lighter than earlier, especially laying down; not thinking about worrisomethoughts, they’re not on my mind; … I feel more relieved in chest/head- no more burden*

(Susan, 39 years old).

Relaxation of the mind and body can help regulate the cognitive–emotional function and minimize “toxic stress”, physiological stress often associated with intrusive symptoms of PTSD [19,21]. As one woman who practiced breathing exercises and mindfulness on her walks shared:

*I feel very relaxed… needed a break from work and the kids; I feel very calm and relaxed; I had a very hectic couple of weeks. This was helpful… reduction sessions helped me calm down, which is something I need now*

(Briana, 32 years old).

#### 3.3.2. Self-Care and Reflection, including Faith-Based Practices

Twelve women (*n* = 12) shared that mindfulness sessions enhanced their motivation towards self-care. The sessions helped them realize the importance of health and happiness in their daily lives. The act of giving back to themselves validated their own worth, especially when many IPV survivors feel as though abuse took something away from them. These small practices of self-management are also good daily coping mechanisms to counteract the cumulative stress that could be toxic to mental health [21]. Women reported reduced stress when practicing self-care activities. One woman highlighted the importance of self-care in her life despite her busy schedule:

*I liked how I can take some time and do these mindfulness activities; Usually I get very stressed and tension builds up with my work emails. I take deep breaths and do some breathing exercises which helps me when I am in my thoughts. Pleasurable activities for me would be to exercise, be more active, go on walks*

(Briana, 32 years old).

For another woman, her self-care practices included self-reflection by taking the time to journal:

*I actually followed your advice and started to write it out. It helps like crazy, but at first, a little awkward… Once you start writing it feels really good*

(Libby, 31 years old).

Faith was a significant part of some women’s lives and beliefs, especially since it proved to be a stronghold during times of trauma. The sessions encouraged the women to continue faith practices, as it helped in stress reduction. These practices included praying (or being present with God), journaling, or meditation of Scripture. One woman survivor of the Christian faith said that she regularly prays and meditates, is her chosen way to cope through difficult circumstances:

*I’ve been doing more of other meditation, before you called, that is one of the things I’ve done and was journaling. I take a scripture, think through it quiet myself and get to the spirit behind that space. I just take my time, so far about 30 min, sometimes an hour*

(Ruth, 30 years old).

#### 3.3.3. Self-Awareness

Self-awareness of body and mind was identified as another helpful component of mindfulness practices. Focusing on sensations and consciousness at the present moment helped strengthen attention control or cognitive–emotion regulation, thereby reducing physiological and mental stress related to IPV [19,21]. Dissociative symptoms were also targeted and mitigated by increased connection to the self and a greater awareness of internal and external experiences [9]. Overall, women shared positive feedback in being more in-tune and present with their bodies and minds. Five women (*n* = 5) reported feeling certain sensations in some areas of their bodies, in their breathing, or in their thoughts.

*Mindful sessions help when mind is wandering everywhere. It helps me calm down and make me aware of the environment. I really needed that today; the body scan audio made me focus on my breathing*

(Briana, 32 years old).

#### 3.3.4. Self-Control and Focused Thinking

The mindfulness phone sessions introduced different stress reduction techniques (e.g., “STOP” or alternative breathing) that encouraged them (*n* = 8) to take control over how they responded to daily stresses in life. Women shared that they were able to first recognize their ability to focus their thinking without distraction and use self-control by not letting the feelings of the stressors control them. These are good practices for strengthening reappraisal, which further reinforces sustained focused attention on unfolding present experiences. Cognitive reappraisal decreases dissociative or ruminating symptoms often found in survivors of IPV and can lead to positive mental health outcomes [19,21]. One woman who regularly practiced “STOP” (a technique taught to re-evaluate the stressful situation before responding to it) when disciplining her children and enjoyed helping people in her spare time, said:

*STOP is like a solution within your reach; STOP, it’s easy to remember. I should use it everyday with my children; I tried it to take my mind away while doing things that I enjoy doing*

(Charlene, age unknown).

Another survivor shared:

*My head isn’t as scattered as before. Before, my brain was thinking many things at a time, but now I am thinking calmly and slowly*

(Sara, 38 years old).

## 4. Discussion

Women with recent IPV experiences in BSHAPE reported positive outcomes after participating in mindfulness sessions. The findings are in line with our quantitative analysis of data [25] where women who participated in the BSHAPE intervention reported significant reduction in stress, improved ability to manage stress, reduced PTSD and depression symptoms, and overall empowerment at post-intervention [25]. Based on the qualitative feedback on the mindfulness component of BSHAPE, we found that mindfulness activities were beneficial in developing positive appraisals and adaptive coping among survivors of IPV. Women highlighted some helpful elements of BSHAPE mindfulness sessions. These included: lovingkindness, self-actualization, intentionality, positivity, relaxation, enhanced motivation towards self-care or reflection, self-awareness, and self-control or focused thinking. Mindfulness encourages the individual to focus on coping strategies and problem solving without having to focus on triggering trauma-related content by sharpening trauma-related cognitions with purposeful acceptance and decentering [9,19].

Research shows that IPV often erodes women’s self-esteem or confidence about their own personal value and worth [8,34]. Thus, interventions are needed to enhance their self-esteem and confidence, and promote self-empowerment [8]. The strategies include helping women understand the effects of trauma on their health and well-being, addressing their negative beliefs or unhealthy coping mechanisms, encouraging personal empowerment and establishing safety in an individualized approach. Women in our study expressed feelings of self-worth as an outcome of engaging in mindfulness practices, as opposed to apathy or negative beliefs about oneself. Mindfulness meditation can teach empathy skills or sensitivity toward oneself and others while cultivating an attitude of love and compassion among survivors of interpersonal violence [18]. With these mindsets and self-actualizations, women are encouraged and self-empowered to move forward in their lives with a renewed positive sense of purpose and self-confidence, while overcoming personal barriers and negative self-perceptions.

In our study, mindfulness activities (e.g., body scan and meditations) stimulated the relaxation and self-awareness of women’s physical and mental states, and further encouraged them to adopt activities of daily care into their lives to better manage stress. This reinforces health benefits to the body and mind while mitigating the debilitating impacts of toxic stress from IPV [18,19,21]. Additionally, women reported feeling encouraged using individualized strategies, such as engaging in faith-based practices or adopting a more hopeful and intentional paradigm of life. This is invaluable for survivors living with PTSD, as it strengthens their coping mechanisms and provides opportunity for positive change, even when they cannot change their external circumstances [16]. Women in our study not only shared having more self-control in their responses to stress, but also indicated using cognitive restructuring strategies (i.e., reappraisal of experiences) without being distracted by prehistoric triggering stressful experiences. This is supported by other research that identified the positive impacts of mindfulness meditation on cognitive attention and concentration in addition to motivational drives [18]. Furthermore, in line with research on women survivors of violence [8,17,21,25], self-empowerment was an overarching theme. In a recent systematic review and meta-analysis of interventions for survivors of IPV, empowerment was found to play a vital role in improving PTSD and depressive symptoms among survivors [34]. Thus, empowerment is a key component of intervention for survivors of IPV with PTSD and/or depressive symptoms.

### 4.1. Recommendations of Mindfulness in the Clinical, Non-Clinical, and Faith-Based Settings

BSHAPE is a culturally tailored and trauma-informed intervention that could be well-applied in diverse clinical and non-clinical settings. According to Borrell-Carrió et al. (2004), a holistic response to a patient’s suffering considers the biological, psychological, and social dimensions of the patient, individualizes the patient’s situation, and gives the patient “a sense of being understood”, while establishing a therapeutic and trusting relationship between the client and the provider [35] (p. 576). This holistic, therapeutic response is especially important for underserved and marginalized racial minorities with a complex history of trauma and IPV [15]. Our feasibility and acceptability evaluation identified the need for additional tailoring of the mindfulness practices to survivor’s needs [25]. Care should be individualized to the woman while prioritizing and supporting her goals in creating a safety plan that she would be able to follow-up with [8,25,35,36]. This not only sets realistic and attainable personal goals for the client, but further empowers her in her decision making and life, while further strengthening her coping mechanisms and healing.

Phone-based mindfulness interventions may be an appropriate method of communication between the facilitator and IPV survivor, as it allows the woman convenience and privacy in her own home without feeling as though she is being the subject under examination. It also encourages a woman to communicate without judgement [25]. There is also compelling evidence that MBSR works, but it cannot work in a vacuum [8,17,18,21,34]. For example, one study recommended providing logistical support with childcare, food, and transport in the MBSR intervention [21], in addition to psychoeducation resources [8,17,21,34]. Mindfulness can be integrated in diverse treatment settings, such as at a substance abuse treatment facility for women [18]. In addition to mindfulness, the BSHAPE intervention integrates strengths-based assessments, feedback, psychoeducation, safety planning, and referrals. These integrated components are needed to assess and strengthen effective coping skills for stressors in women’s daily lives, empowering them step by step, and building long-lasting resilience [37]. Remote mindfulness practices can be beneficial for women immigrant survivors of IPV who face numerous barriers, such as stigmatization and language, in accessing services. Especially in non-mental health settings, mindfulness practices can be implemented in a cost-effective manner, making it more acceptable and feasible for underserved and marginalized populations [18,21], such as Black immigrant women survivors of IPV [25].

Some women in BSHAPE expressed a faith-based background (e.g., Christianity or Islam) in which they regularly practiced and congregated with others of the same faith. Contrary to hesitant perceptions of Christianity or other religions toward Buddhist-associated practices, mindfulness may prove to be a beneficial component of faith-based therapies. It could even strengthen certain values (e.g., forgiveness and compassion) and practices of faith (e.g., cultivating a life of mindful prayer, being present with God, or having a posture of gratitude or awe and wonder toward the beauty of life) without further compromising core beliefs and values [25,33]. Thus, such practices could be linked to faith-based beliefs when working with survivors for whom faith is a fundamental part of their lives.

### 4.2. Recommendations for Further Research

Most studies to date for IPV survivors examined the impact of in-person individual or group-based therapeutic approaches, such as cognitive behavioral therapy. Additional research is needed on the usefulness of remote, individual, and cost-effective approaches to care for immigrant women survivors of IPV who face numerous barriers to accessing standard care services. Studies are needed to examine if mindfulness can be effective as a stand-alone intervention or in combination with other mental health therapeutic approaches.

### 4.3. Limitations

There were several limitations to this study. This preliminary evaluation and feedback is based on a relatively small sample of IPV survivors. Additional research using a large sample size of diverse groups of immigrant survivors of IPV can strengthen our study findings. The feedback given by the women was self-reported, which may have biased the results. Although the phone calls were more feasible in a pandemic setting due to COVID-19, they were less conducive to a setting where women were multi-tasking or were in a distractive environment. Only English speakers were included in this small pilot study, which limited the generalizability of the study’s findings to non-English speaking immigrant women in the United States. The next iteration of the BSHAPE trial will incorporate multiple languages and more tailored approaches with diverse groups of immigrant survivors of IPV.

## 5. Conclusions

Immigrant women with IPV experiences in the US face an understated number of external obstacles and internal suffering, which negatively affect their mental health and well-being. Culturally sensitive mindfulness practices may help alleviate women’s daily physical and mental stress, as well as enhance positive coping and empowerment. Mindfulness practices can offer women an opportunity to reappraise their own lives during stressful experiences by equipping them with the tools and skills they need to overcome and heal in a way that is meaningful to them.

## Data Availability

The data generated for this article is presented in the results of the study.
